# Crystal structure of zwitterionic 2-[bis­(2-meth­oxy­phen­yl)phosphanium­yl]-4-methyl­benzene­sulfonate monohydrate di­chloro­methane monosolvate

**DOI:** 10.1107/S2056989016000669

**Published:** 2016-01-27

**Authors:** Hongyang Zhang, Ge Feng, Alexander S. Filatov, Richard F. Jordan

**Affiliations:** a Department of Chemistry, the University of Chicago, 5735 South Ellis ave, Chicago, IL 60637, USA

**Keywords:** crystal structure, sulfonic acid, zwitterion, hydrogen bonding

## Abstract

In the title compound, C_21_H_21_O_5_PS·H_2_O·CH_2_Cl_2_, the phospho­nium–sulfonate zwitterion has the acidic H atom located on the P atom rather than the sulfonate group. The S—O bond lengths [1.4453 (15)–1.4521 (14) Å] are essentially equal. In the crystal, the water mol­ecules bridge two zwitterions *via* O_water_—H⋯O_sulfonate_ hydrogen bonds into a centrosymmetric dimer. The dimers are further linked by weak C_Ar­yl_—H⋯O_sulfonate_ hydrogen bonds into chains extending along [100]. The PH^+^ group is not involved in inter­molecular inter­actions.

## Abstract 2

The phospho­nium–sulfonate zwitterion has the acidic H atom located on the P atom rather than the sulfonate group. The PH^+^ group is not involved in inter­molecular inter­actions.

## Chemical context   

Phosphane ligands (Allen, 2014 ▸) are ubiquitous in coordination and organometallic chemistry and have been used to synthesize a wide variety of metal complexes and catalysts (Hartwig, 2010 ▸). Incorporation of additional potential donor groups within the phosphane structure provides added versatility to such ligands. For example, *ortho*-phosphanyl-benzene­sulfonate (PO) ligands, such as 2-[bis­(2-meth­oxy­phen­yl)phosphanyl]benzene­sulfonate, bind to Pd^II^ in a κ^2^ *P,O* mode to form (PO)Pd*R* species that are active for the polymerization of ethyl­ene (Cai *et al.*, 2012 ▸; Contrella & Jordan, 2014 ▸; Zhou *et al.*, 2014 ▸), copolymerization of ethyl­ene and polar monomers (Drent *et al.*, 2002**a* ▸;* Nakamura *et al.*, 2013 ▸), non-alternating copolymerization of ethyl­ene and CO (Drent *et al.*, 2002*b* ▸), and alternating copolymerization of CO with polar monomers (Nakamura *et al.*, 2011 ▸, 2012 ▸). Phosphanyl-arene­sulfonate ligands derived from *para*-toluene­sulfonic acid are useful because the extra methyl group provides a convenient NMR handle for characterizing complexes and monitoring reactions.
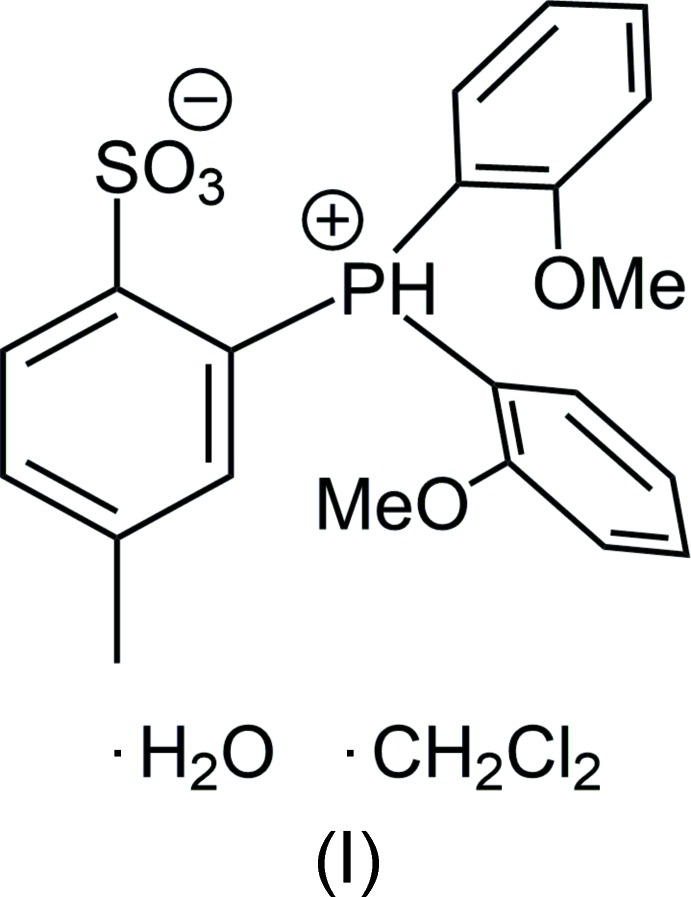


The zwitterion 2-[bis­(2-meth­oxy­phen­yl)phosphanium­yl]-4-methyl­benzene­sulfonate (**1**, Scheme 1) was synthesized by sequential reaction of PCl_3_ with dili­thia­ted *p*-tol­uene­sulfonate and 1-li­thio-2-meth­oxy­benzene, followed by acidification of HCl (Scheme 2) (Vela *et al.*, 2007 ▸). Here we report the crystal structure of **1**·H_2_O·CH_2_Cl_2_, (I).
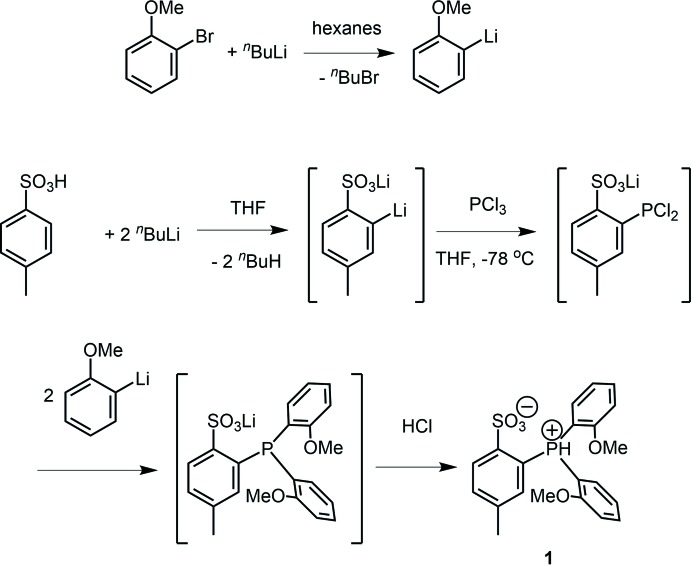


## Structural commentary   

**Figure 1:**
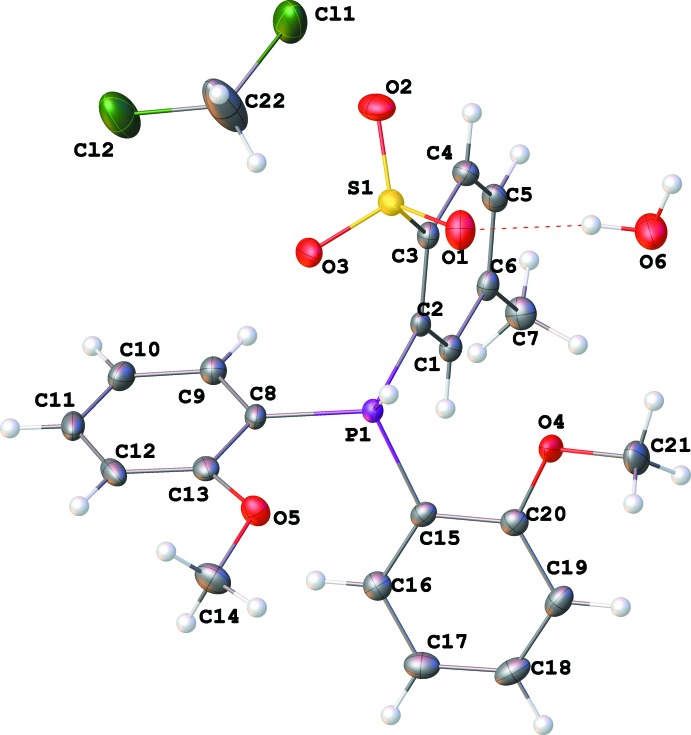
The mol­ecular structure of the title compound, showing the atom labelling. Displacement ellipsoids are drawn at the 50% probability level. The dashed line denotes a hydrogen bond.

Compound **1** crystallizes as the phospho­nium–sulfonate zwitterion in which the acidic H atom is located on the P atom rather than the sulfonate group (Fig. 1 ▸). The S—O bond distances fall within the narrow range of 1.4453 (15) to 1.4521 (14) Å, and the P—C distances lie within the range of 1.7794 (18) to 1.7984 (18) Å. The P—H atom was located in a difference Fourier map and refined without additional restraints. The P—H bond length is 1.22 (2) Å. Compound **1** adopts an *exo*_3_ conformation, *i.e.* the *ortho* meth­oxy and sulfonate groups point toward the PH^+^ group (Feng *et al.*, 2014 ▸). Tris(*ortho*-substituted ar­yl)phosphanes normally exhibit *exo*_3_ conformations (Howell *et al.*, 1999 ▸) because the *ortho* substituents cause less steric congestion when they point toward the P lone pair (*exo*) rather than toward the other aryl rings (*endo*). Addition of an H^+^ at phospho­rous should not add significant steric congestion and therefore it is not surprising that **1** also adopts the *exo*_3_ conformation. The O_meth­oxy_⋯P distances, 2.7691 (14) and 2.7940 (14) Å, are shorter than the sum of the O and P van der Waals radii (3.35 Å). The O3⋯H1(P1) distance is 2.44 (2) Å.

## DFT calculations   

**Figure 2:**
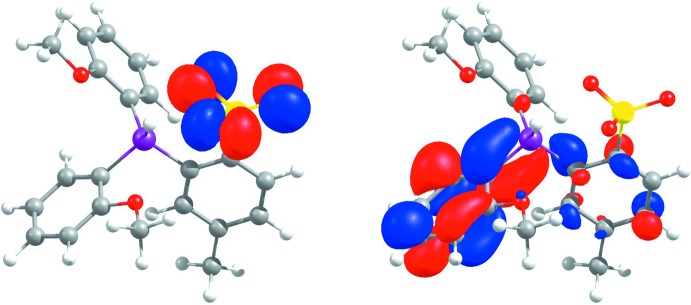
HOMO (−0.2289 Hartrees, left) and LUMO (−0.0483 Hartrees, right) orbitals of **1**.

The relative stability of the observed *exo*_3_ conformation *versus* alternative *exo*_2_ and *exo*_1_ conformations was investigated by DFT calculations using the hybrid exchange-correlation functional PBE0 (Perdew *et al.*, 1996 ▸, 1997 ▸) and the 6-311G(d,p) basis set for all atoms. The optimized structure is the *exo*_3_ conformer, in which the meth­oxy and sulfonate groups point toward the PH^+^ group. Geometry optimizations were also carried out on two conformers in which the SO_3_ group was kept *exo* but one (*exo*_2_) or two (*exo*_1_) meth­oxy groups were rotated away from the PH^+^ group. The *exo*_2_ and *exo*_1_ conformers were calculated to be 1.2 and 2.5 kcal mol^−1^ less stable than the *exo*_3_ isomer, respectively. The HOMO of the *exo*_3_ conformer is comprised of *p* orbitals of the sulfonate O atoms, while the LUMO is delocalized over the phenyl rings and P—C_aromatic_ bonds (Fig. 2 ▸).

## Supra­molecular features   

**Figure 3:**
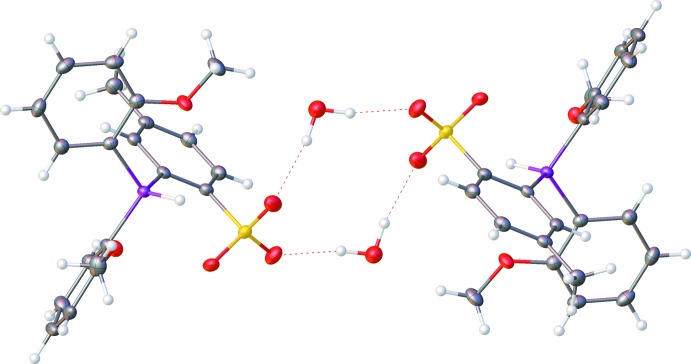
Dimer formation through O_water_—H⋯O_sulfonate_ hydrogen bonds (dashed lines).

**Figure 4:**
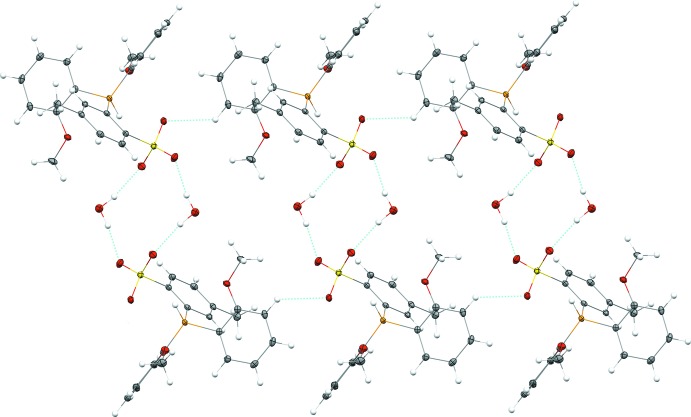
A fragment of the crystal packing of the title compound with inter­molecular hydrogen bonds shown as dashed light-blue lines. Color scheme: C grey, H white, O red, P orange, S yellow.

Two O atoms of the SO_3_^−^ group are hydrogen bonded with the co-crystallized water mol­ecule, forming inversion dimers (Fig. 3 ▸). The O_water_—H⋯O_sulfonate_ contacts are 1.96 (3) and 1.98 (3) Å (Table 1 ▸). These dimers are further linked by C_Aryl_—H⋯O_sulfonate_ hydrogen bonds into infinite chains running along the [100] direction (Fig. 4 ▸). A similar C_Ar_–SO_3_^−^⋯H_2_O⋯C_Ar_–SO_3_^−^⋯H_2_O⋯ hydrogen-bonding motif was observed in [Na(18-crown-6)(H_2_O)][2-{(*o*-CF_3_-Ph)_2_P}-4-Me-benzene­sulfonate] (Feng *et al.*, 2014 ▸).

## Database survey   

A search of the Cambridge Structural Database (CSD, Version 5.36, last update May 2015; Groom & Allen, 2014 ▸) revealed structural reports for two analogues of **1** that contain 4-chloro-substituted meth­oxy­phenyl (CSD refcode ODUNOS; Wucher *et al.*, 2013 ▸) or 2,6-di­meth­oxy­phenyl substituents at phospho­rous (CSD refcode: LEXLEG; Liu *et al.*, 2007 ▸). These compounds also crystallized as zwitterions in which the acidic proton is located on the P atom and feature close O_meth­oxy_⋯P contacts (2.764 to 2.927 Å). The structure of the tri­ethyl­ammonium salt of 2-[bis­(2-meth­oxy­phen­yl)phos­phanyl]benzene­sulfonate has also been reported (CSD refcode HAGKEH; Bettucci *et al.*, 2008 ▸). In this case, the acidic H atom is located at tri­ethyl­amine rather than on the P atom and the O_meth­oxy_⋯P distances are 2.877 and 2.903 Å.

## Synthesis and crystallization   

Compound **1** was synthesized by a modification of a previously reported procedure (Vela *et al.*, 2007 ▸) comprising sequential reaction of PCl_3_ with dili­thia­ted *p*-toluene­sulfonate and 1-li­thio-2-meth­oxy­benzene, followed by acidification of HCl, to afford **1** in 70–75% yield on a 3–4 g scale (Scheme 2). The product was purified by recrystallization (CH_2_Cl_2_/Et_2_O, volume ratio 1/3, layering at 273K). Crystals of **1**·H_2_O·CH_2_Cl_2_ (**I**) suitable for the X-ray diffraction analysis were obtained by layering Et_2_O on a CH_2_Cl_2_ solution of **1** at 277 K.

## Refinement   

Crystal data, data collection and structure refinement details are summarized in Table 2 ▸. Carbon-bound H atoms were placed in calculated positions (C—H = 0.95–0.98 Å) and were included in the refinement in the riding-model approximation, with *U*_iso_(H) set to 1.2–1.5*U*_eq_(C). The P- and O-bound H atoms were located in a difference Fourier map and refined isotropically.

## Supplementary Material

Crystal structure: contains datablock(s) I. DOI: 10.1107/S2056989016000669/cv5502sup1.cif

Structure factors: contains datablock(s) I. DOI: 10.1107/S2056989016000669/cv5502Isup2.hkl

Supporting information file. DOI: 10.1107/S2056989016000669/cv5502Isup3.cml

CCDC reference: 1447138

Additional supporting information:  crystallographic information; 3D view; checkCIF report

## Figures and Tables

**Table 1:** Hydrogen-bond geometry (Å, °)

*D*—H⋯*A*	*D*—H	H⋯*A*	*D*⋯*A*	*D*—H⋯*A*
O6—H1*O*⋯O1	0.91 (3)	1.96 (3)	2.862 (2)	170 (3)
O6—H2*O*⋯O2^i^	0.92 (3)	1.98 (3)	2.877 (2)	164 (3)
C19—H19⋯O3^ii^	0.95	2.47	3.180 (2)	132

Symmetry codes: (i) 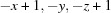; (ii) 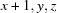.

**Table 2:** Experimental details

Crystal data	
Chemical formula	C_21_H_21_O_5_PS·CH_2_Cl_2_·H_2_O
*M*_r_	519.35
Crystal system, space group	Monoclinic, *P*2_1_/*n*
Temperature (K)	100
*a*, *b*, *c* (Å)	9.6437 (6), 15.9441 (11), 15.9641 (11)
β (°)	105.051 (2)
*V* (Å^3^)	2370.4 (3)
*Z*	4
Radiation type	Mo *K*α
μ (mm^−1^)	0.47
Crystal size (mm)	0.32 × 0.18 × 0.12

Data collection	
Diffractometer	Bruker D8 Venture PHOTON 100 CMOS
Absorption correction	Multi-scan (*SADABS*; Bruker, 2014 ▸)
*T*_min_, *T*_max_	0.693, 0.745
No. of measured, independent and observed [*I* > 2σ(*I*)] reflections	53574, 4888, 4349
*R*_int_	0.030
(sin θ/λ)_max_ (Å^−1^)	0.627

Refinement	
*R*[*F* ^2^ > 2σ(*F* ^2^)], *wR*(*F* ^2^), *S*	0.038, 0.106, 1.05
No. of reflections	4888
No. of parameters	304
H-atom treatment	H atoms treated by a mixture of independent and constrained refinement
Δρ_max_, Δρ_min_ (e Å^−3^)	0.48, −0.66

Computer programs: *APEX2* and *SAINT* (Bruker, 2014 ▸), *SHELXT* (Sheldrick, 2015*a* ▸), *SHELXL2014* (Sheldrick, 2015*b* ▸), *OLEX2* (Dolomanov *et al.*, 2009 ▸), *Mercury* (Macrae *et al.*, 2008 ▸) and *publCIF* (Westrip, 2010 ▸).

## supplementary crystallographic information

### Crystal data

**Table:**

C_21_H_21_O_5_PS·CH_2_Cl_2_·H_2_O	*F*(000) = 1080
*M**_r_* = 519.35	*D*_x_ = 1.455 Mg m^−^^3^
Monoclinic, *P*2_1_/*n*	Mo *K*α radiation, λ = 0.71073 Å
*a* = 9.6437 (6) Å	Cell parameters from 9610 reflections
*b* = 15.9441 (11) Å	θ = 2.2–26.4°
*c* = 15.9641 (11) Å	µ = 0.47 mm^−^^1^
β = 105.051 (2)°	*T* = 100 K
*V* = 2370.4 (3) Å^3^	Block, colorless
*Z* = 4	0.32 × 0.18 × 0.12 mm

### Data collection

**Table:**

Bruker D8 Venture PHOTON 100 CMOS diffractometer	4888 independent reflections
Radiation source: INCOATEC ImuS micro-focus source	4349 reflections with *I* > 2σ(*I*)
Mirrors monochromator	*R*_int_ = 0.030
Detector resolution: 10.4167 pixels mm^-1^	θ_max_ = 26.5°, θ_min_ = 2.2°
ω and phi scans	*h* = −12→12
Absorption correction: multi-scan (*SADABS*; Bruker, 2014)	*k* = −19→19
*T*_min_ = 0.693, *T*_max_ = 0.745	*l* = −20→19
53574 measured reflections	

### Refinement

**Table:**

Refinement on *F*^2^	Primary atom site location: dual
Least-squares matrix: full	Secondary atom site location: difference Fourier map
*R*[*F*^2^ > 2σ(*F*^2^)] = 0.038	Hydrogen site location: mixed
*wR*(*F*^2^) = 0.106	H atoms treated by a mixture of independent and constrained refinement
*S* = 1.05	*w* = 1/[σ^2^(*F*_o_^2^) + (0.0536*P*)^2^ + 2.7024*P*] where *P* = (*F*_o_^2^ + 2*F*_c_^2^)/3
4888 reflections	(Δ/σ)_max_ = 0.001
304 parameters	Δρ_max_ = 0.48 e Å^−^^3^
0 restraints	Δρ_min_ = −0.66 e Å^−^^3^

### Special details

**Table:**

Geometry. All e.s.d.'s (except the e.s.d. in the dihedral angle between two l.s. planes) are estimated using the full covariance matrix. The cell e.s.d.'s are taken into account individually in the estimation of e.s.d.'s in distances, angles and torsion angles; correlations between e.s.d.'s in cell parameters are only used when they are defined by crystal symmetry. An approximate (isotropic) treatment of cell e.s.d.'s is used for estimating e.s.d.'s involving l.s. planes.

### Fractional atomic coordinates and isotropic or equivalent isotropic displacement parameters (Å^2^)

**Table:**

	*x*	*y*	*z*	*U*_iso_*/*U*_eq_	
P1	0.63863 (5)	0.30456 (3)	0.33309 (3)	0.01288 (12)	
H1P	0.592 (2)	0.2397 (14)	0.2950 (14)	0.018 (5)*	
S1	0.43769 (5)	0.17697 (3)	0.41453 (3)	0.01702 (12)	
O1	0.50338 (15)	0.10620 (9)	0.38266 (10)	0.0261 (3)	
O2	0.34252 (15)	0.15453 (10)	0.46751 (10)	0.0269 (3)	
O3	0.37411 (14)	0.23600 (9)	0.34604 (9)	0.0210 (3)	
O4	0.86431 (15)	0.19197 (8)	0.35798 (9)	0.0210 (3)	
O5	0.51406 (15)	0.34403 (9)	0.15930 (8)	0.0206 (3)	
C1	0.77958 (19)	0.33171 (11)	0.50245 (12)	0.0155 (4)	
H1	0.8372	0.3678	0.4781	0.019*	
C2	0.66711 (19)	0.28789 (11)	0.44773 (11)	0.0139 (3)	
C3	0.58188 (19)	0.23434 (11)	0.48294 (12)	0.0155 (4)	
C4	0.6117 (2)	0.22566 (12)	0.57215 (12)	0.0188 (4)	
H4	0.5552	0.1890	0.5966	0.023*	
C5	0.7235 (2)	0.27025 (12)	0.62605 (12)	0.0187 (4)	
H5	0.7417	0.2642	0.6871	0.022*	
C6	0.8093 (2)	0.32358 (12)	0.59237 (12)	0.0170 (4)	
C7	0.9324 (2)	0.37081 (13)	0.65044 (13)	0.0227 (4)	
H7A	0.9220	0.3708	0.7099	0.034*	
H7B	0.9323	0.4287	0.6299	0.034*	
H7C	1.0231	0.3437	0.6493	0.034*	
C8	0.51505 (19)	0.38810 (11)	0.29746 (12)	0.0159 (4)	
C9	0.4711 (2)	0.44064 (12)	0.35526 (13)	0.0202 (4)	
H9	0.5089	0.4336	0.4160	0.024*	
C10	0.3720 (2)	0.50321 (13)	0.32356 (14)	0.0239 (4)	
H10	0.3426	0.5402	0.3624	0.029*	
C11	0.3158 (2)	0.51167 (13)	0.23483 (15)	0.0246 (4)	
H11	0.2461	0.5540	0.2136	0.030*	
C12	0.3583 (2)	0.46027 (12)	0.17634 (13)	0.0209 (4)	
H12	0.3185	0.4670	0.1157	0.025*	
C13	0.46023 (19)	0.39848 (12)	0.20784 (12)	0.0173 (4)	
C14	0.4860 (2)	0.36077 (14)	0.06819 (13)	0.0261 (4)	
H14A	0.3827	0.3561	0.0413	0.039*	
H14B	0.5378	0.3201	0.0416	0.039*	
H14C	0.5184	0.4176	0.0595	0.039*	
C15	0.80656 (19)	0.32846 (12)	0.31142 (11)	0.0152 (4)	
C16	0.8366 (2)	0.40458 (12)	0.27736 (12)	0.0192 (4)	
H16	0.7672	0.4482	0.2665	0.023*	
C17	0.9686 (2)	0.41647 (13)	0.25930 (13)	0.0224 (4)	
H17	0.9894	0.4678	0.2349	0.027*	
C18	1.0697 (2)	0.35248 (13)	0.27735 (13)	0.0226 (4)	
H18	1.1612	0.3615	0.2670	0.027*	
C19	1.0407 (2)	0.27600 (13)	0.30998 (13)	0.0208 (4)	
H19	1.1106	0.2326	0.3208	0.025*	
C20	0.9083 (2)	0.26368 (12)	0.32663 (12)	0.0173 (4)	
C21	0.9467 (2)	0.11736 (13)	0.35617 (14)	0.0258 (4)	
H21A	0.9509	0.1062	0.2965	0.039*	
H21B	0.9012	0.0699	0.3775	0.039*	
H21C	1.0442	0.1251	0.3932	0.039*	
C22	0.2653 (5)	0.3457 (2)	0.5203 (2)	0.0676 (11)	
H22A	0.2056	0.3047	0.4801	0.081*	
H22B	0.3569	0.3518	0.5038	0.081*	
Cl1	0.30192 (7)	0.30568 (5)	0.62409 (4)	0.04587 (19)	
Cl2	0.17791 (9)	0.44136 (4)	0.50619 (5)	0.0517 (2)	
O6	0.73753 (17)	−0.00322 (11)	0.45863 (11)	0.0308 (4)	
H1O	0.656 (4)	0.027 (2)	0.436 (2)	0.049 (8)*	
H2O	0.701 (3)	−0.053 (2)	0.4720 (19)	0.043 (8)*	

### Atomic displacement parameters (Å^2^)

**Table:**

	*U*^11^	*U*^22^	*U*^33^	*U*^12^	*U*^13^	*U*^23^
P1	0.0121 (2)	0.0138 (2)	0.0124 (2)	0.00178 (16)	0.00263 (17)	0.00083 (16)
S1	0.0143 (2)	0.0180 (2)	0.0173 (2)	−0.00200 (17)	0.00148 (17)	0.00157 (17)
O1	0.0245 (7)	0.0214 (7)	0.0283 (8)	0.0011 (6)	−0.0007 (6)	−0.0049 (6)
O2	0.0227 (7)	0.0324 (8)	0.0255 (8)	−0.0092 (6)	0.0062 (6)	0.0041 (6)
O3	0.0141 (6)	0.0255 (7)	0.0207 (7)	−0.0009 (5)	−0.0003 (5)	0.0048 (6)
O4	0.0217 (7)	0.0174 (7)	0.0268 (7)	0.0068 (5)	0.0115 (6)	0.0045 (5)
O5	0.0244 (7)	0.0213 (7)	0.0148 (7)	0.0010 (6)	0.0027 (5)	0.0006 (5)
C1	0.0151 (8)	0.0143 (8)	0.0173 (9)	0.0025 (7)	0.0044 (7)	−0.0010 (7)
C2	0.0147 (8)	0.0138 (8)	0.0134 (8)	0.0043 (7)	0.0043 (7)	0.0012 (7)
C3	0.0137 (8)	0.0159 (9)	0.0162 (9)	0.0026 (7)	0.0025 (7)	0.0006 (7)
C4	0.0182 (9)	0.0209 (9)	0.0179 (9)	0.0014 (7)	0.0058 (7)	0.0035 (7)
C5	0.0213 (9)	0.0210 (9)	0.0139 (9)	0.0045 (7)	0.0047 (7)	0.0015 (7)
C6	0.0160 (9)	0.0168 (9)	0.0173 (9)	0.0047 (7)	0.0026 (7)	−0.0020 (7)
C7	0.0234 (10)	0.0239 (10)	0.0186 (9)	−0.0010 (8)	0.0017 (8)	−0.0030 (8)
C8	0.0123 (8)	0.0153 (9)	0.0198 (9)	0.0020 (7)	0.0038 (7)	0.0036 (7)
C9	0.0198 (9)	0.0202 (10)	0.0211 (9)	0.0029 (7)	0.0063 (8)	0.0025 (7)
C10	0.0229 (10)	0.0199 (10)	0.0323 (11)	0.0051 (8)	0.0131 (9)	0.0028 (8)
C11	0.0159 (9)	0.0206 (10)	0.0381 (12)	0.0039 (7)	0.0080 (8)	0.0117 (9)
C12	0.0162 (9)	0.0209 (9)	0.0229 (10)	−0.0024 (7)	0.0002 (7)	0.0093 (8)
C13	0.0133 (8)	0.0172 (9)	0.0211 (9)	−0.0029 (7)	0.0039 (7)	0.0027 (7)
C14	0.0339 (11)	0.0273 (11)	0.0166 (9)	−0.0028 (9)	0.0055 (8)	0.0023 (8)
C15	0.0136 (8)	0.0191 (9)	0.0131 (8)	−0.0004 (7)	0.0037 (7)	−0.0023 (7)
C16	0.0199 (9)	0.0189 (9)	0.0183 (9)	−0.0006 (7)	0.0040 (7)	−0.0011 (7)
C17	0.0247 (10)	0.0223 (10)	0.0213 (10)	−0.0082 (8)	0.0077 (8)	−0.0020 (8)
C18	0.0170 (9)	0.0303 (11)	0.0221 (10)	−0.0056 (8)	0.0083 (8)	−0.0079 (8)
C19	0.0158 (9)	0.0272 (10)	0.0196 (9)	0.0025 (8)	0.0049 (7)	−0.0045 (8)
C20	0.0183 (9)	0.0196 (9)	0.0140 (8)	−0.0001 (7)	0.0042 (7)	−0.0015 (7)
C21	0.0311 (11)	0.0198 (10)	0.0289 (11)	0.0109 (8)	0.0117 (9)	0.0024 (8)
C22	0.126 (3)	0.0495 (18)	0.0368 (15)	0.043 (2)	0.0374 (19)	0.0161 (13)
Cl1	0.0453 (4)	0.0621 (4)	0.0305 (3)	0.0225 (3)	0.0103 (3)	0.0118 (3)
Cl2	0.0798 (5)	0.0347 (3)	0.0452 (4)	0.0190 (3)	0.0245 (4)	0.0098 (3)
O6	0.0241 (8)	0.0272 (8)	0.0389 (9)	−0.0020 (7)	0.0040 (7)	0.0056 (7)

### Geometric parameters (Å, º)

**Table:**

P1—C8	1.7794 (18)	C10—C11	1.386 (3)
P1—C15	1.7828 (18)	C10—H10	0.9500
P1—C2	1.7984 (18)	C11—C12	1.382 (3)
P1—H1P	1.22 (2)	C11—H11	0.9500
S1—O2	1.4453 (15)	C12—C13	1.390 (3)
S1—O1	1.4495 (15)	C12—H12	0.9500
S1—O3	1.4521 (14)	C14—H14A	0.9800
S1—C3	1.7816 (19)	C14—H14B	0.9800
O4—C20	1.359 (2)	C14—H14C	0.9800
O4—C21	1.435 (2)	C15—C16	1.391 (3)
O5—C13	1.354 (2)	C15—C20	1.402 (3)
O5—C14	1.434 (2)	C16—C17	1.389 (3)
C1—C2	1.391 (3)	C16—H16	0.9500
C1—C6	1.395 (3)	C17—C18	1.389 (3)
C1—H1	0.9500	C17—H17	0.9500
C2—C3	1.400 (3)	C18—C19	1.383 (3)
C3—C4	1.385 (3)	C18—H18	0.9500
C4—C5	1.388 (3)	C19—C20	1.384 (3)
C4—H4	0.9500	C19—H19	0.9500
C5—C6	1.388 (3)	C21—H21A	0.9800
C5—H5	0.9500	C21—H21B	0.9800
C6—C7	1.505 (3)	C21—H21C	0.9800
C7—H7A	0.9800	C22—Cl1	1.725 (3)
C7—H7B	0.9800	C22—Cl2	1.728 (3)
C7—H7C	0.9800	C22—H22A	0.9900
C8—C9	1.391 (3)	C22—H22B	0.9900
C8—C13	1.400 (3)	O6—H1O	0.91 (3)
C9—C10	1.384 (3)	O6—H2O	0.92 (3)
C9—H9	0.9500		
			
C8—P1—C15	110.16 (9)	C12—C11—C10	121.81 (18)
C8—P1—C2	110.40 (8)	C12—C11—H11	119.1
C15—P1—C2	108.82 (8)	C10—C11—H11	119.1
C8—P1—H1P	110.0 (10)	C11—C12—C13	118.80 (18)
C15—P1—H1P	108.8 (10)	C11—C12—H12	120.6
C2—P1—H1P	108.6 (10)	C13—C12—H12	120.6
O2—S1—O1	114.48 (9)	O5—C13—C12	125.99 (18)
O2—S1—O3	113.14 (9)	O5—C13—C8	114.20 (16)
O1—S1—O3	112.20 (9)	C12—C13—C8	119.81 (18)
O2—S1—C3	106.35 (9)	O5—C14—H14A	109.5
O1—S1—C3	105.80 (8)	O5—C14—H14B	109.5
O3—S1—C3	103.80 (8)	H14A—C14—H14B	109.5
C20—O4—C21	117.55 (15)	O5—C14—H14C	109.5
C13—O5—C14	117.51 (15)	H14A—C14—H14C	109.5
C2—C1—C6	121.17 (17)	H14B—C14—H14C	109.5
C2—C1—H1	119.4	C16—C15—C20	120.20 (17)
C6—C1—H1	119.4	C16—C15—P1	123.67 (14)
C1—C2—C3	119.83 (17)	C20—C15—P1	116.05 (14)
C1—C2—P1	116.93 (14)	C17—C16—C15	119.71 (18)
C3—C2—P1	123.24 (14)	C17—C16—H16	120.1
C4—C3—C2	119.16 (17)	C15—C16—H16	120.1
C4—C3—S1	119.96 (14)	C16—C17—C18	119.23 (19)
C2—C3—S1	120.87 (14)	C16—C17—H17	120.4
C3—C4—C5	120.43 (18)	C18—C17—H17	120.4
C3—C4—H4	119.8	C19—C18—C17	121.78 (18)
C5—C4—H4	119.8	C19—C18—H18	119.1
C6—C5—C4	121.25 (17)	C17—C18—H18	119.1
C6—C5—H5	119.4	C18—C19—C20	118.94 (18)
C4—C5—H5	119.4	C18—C19—H19	120.5
C5—C6—C1	118.16 (17)	C20—C19—H19	120.5
C5—C6—C7	121.47 (17)	O4—C20—C19	125.57 (18)
C1—C6—C7	120.37 (17)	O4—C20—C15	114.34 (16)
C6—C7—H7A	109.5	C19—C20—C15	120.09 (18)
C6—C7—H7B	109.5	O4—C21—H21A	109.5
H7A—C7—H7B	109.5	O4—C21—H21B	109.5
C6—C7—H7C	109.5	H21A—C21—H21B	109.5
H7A—C7—H7C	109.5	O4—C21—H21C	109.5
H7B—C7—H7C	109.5	H21A—C21—H21C	109.5
C9—C8—C13	120.48 (17)	H21B—C21—H21C	109.5
C9—C8—P1	122.19 (15)	Cl1—C22—Cl2	115.01 (17)
C13—C8—P1	117.32 (14)	Cl1—C22—H22A	108.5
C10—C9—C8	119.47 (19)	Cl2—C22—H22A	108.5
C10—C9—H9	120.3	Cl1—C22—H22B	108.5
C8—C9—H9	120.3	Cl2—C22—H22B	108.5
C9—C10—C11	119.59 (19)	H22A—C22—H22B	107.5
C9—C10—H10	120.2	H1O—O6—H2O	102 (3)
C11—C10—H10	120.2		
			
C6—C1—C2—C3	−0.2 (3)	C8—C9—C10—C11	1.2 (3)
C6—C1—C2—P1	179.37 (14)	C9—C10—C11—C12	−1.4 (3)
C8—P1—C2—C1	−91.04 (15)	C10—C11—C12—C13	0.0 (3)
C15—P1—C2—C1	29.98 (16)	C14—O5—C13—C12	12.9 (3)
C8—P1—C2—C3	88.50 (16)	C14—O5—C13—C8	−167.84 (16)
C15—P1—C2—C3	−150.48 (15)	C11—C12—C13—O5	−179.18 (17)
C1—C2—C3—C4	−0.3 (3)	C11—C12—C13—C8	1.6 (3)
P1—C2—C3—C4	−179.79 (14)	C9—C8—C13—O5	178.92 (16)
C1—C2—C3—S1	−179.67 (13)	P1—C8—C13—O5	−2.3 (2)
P1—C2—C3—S1	0.8 (2)	C9—C8—C13—C12	−1.7 (3)
O2—S1—C3—C4	21.46 (18)	P1—C8—C13—C12	177.04 (14)
O1—S1—C3—C4	−100.67 (16)	C8—P1—C15—C16	4.33 (19)
O3—S1—C3—C4	141.04 (15)	C2—P1—C15—C16	−116.84 (16)
O2—S1—C3—C2	−159.14 (15)	C8—P1—C15—C20	−172.54 (14)
O1—S1—C3—C2	78.73 (16)	C2—P1—C15—C20	66.29 (16)
O3—S1—C3—C2	−39.55 (17)	C20—C15—C16—C17	−0.7 (3)
C2—C3—C4—C5	0.8 (3)	P1—C15—C16—C17	−177.42 (15)
S1—C3—C4—C5	−179.82 (14)	C15—C16—C17—C18	−1.3 (3)
C3—C4—C5—C6	−0.8 (3)	C16—C17—C18—C19	2.3 (3)
C4—C5—C6—C1	0.4 (3)	C17—C18—C19—C20	−1.3 (3)
C4—C5—C6—C7	−178.82 (18)	C21—O4—C20—C19	−14.4 (3)
C2—C1—C6—C5	0.1 (3)	C21—O4—C20—C15	165.79 (17)
C2—C1—C6—C7	179.34 (17)	C18—C19—C20—O4	179.46 (18)
C15—P1—C8—C9	−109.47 (16)	C18—C19—C20—C15	−0.8 (3)
C2—P1—C8—C9	10.75 (19)	C16—C15—C20—O4	−178.46 (16)
C15—P1—C8—C13	71.78 (16)	P1—C15—C20—O4	−1.5 (2)
C2—P1—C8—C13	−168.00 (14)	C16—C15—C20—C19	1.7 (3)
C13—C8—C9—C10	0.3 (3)	P1—C15—C20—C19	178.73 (14)
P1—C8—C9—C10	−178.39 (15)		

### Hydrogen-bond geometry (Å, º)

**Table:**

*D*—H···*A*	*D*—H	H···*A*	*D*···*A*	*D*—H···*A*
O6—H1*O*···O1	0.91 (3)	1.96 (3)	2.862 (2)	170 (3)
O6—H2*O*···O2^i^	0.92 (3)	1.98 (3)	2.877 (2)	164 (3)
C19—H19···O3^ii^	0.95	2.47	3.180 (2)	132

Symmetry codes: (i) −*x*+1, −*y*, −*z*+1; (ii) *x*+1, *y*, *z*.
